# Hypothesis to explain the disparity in the proportion of liver metastases between appendiceal and colorectal cancer

**DOI:** 10.1093/bjs/znab472

**Published:** 2022-02-08

**Authors:** Adam T. Cristaudo, David L. Morris

**Affiliations:** Liver and Peritonectomy Unit, Department of Surgery, St George Hospital, Kogarah, New South Wales, Australia

*Dear Editor*

Within our institution, the authors recently noted a significant disparity in the proportion of liver metastases between appendiceal and colorectal adenocarcinomas (3.1 and 24 per cent respectively). This is consistent with current literature, which describes 2–5 per cent of patients with appendiceal cancer having synchronous liver metastases, as opposed to 25 per cent of those with colorectal cancer^1,2^. The role of carcinoembryonic antigen (CEA) in the production of liver metastases in colorectal cancer has been described in a recent paper by Lee and Lee^3^; CEA produced by colorectal cancers travels via the portal vein to the liver (once venous invasion of the primary tumour has occurred) and binds to CEA receptors within Kupffer cells of the liver. This causes a metastatic cascade of events leading to the formation of liver metastases. As an extrapolation of this, a hypothesis has been proposed by the first author regarding the natural progression of appendiceal cancer.

Appendiceal cancer begins with epithelial proliferation, associated mucin production, and luminal obstruction, followed by either perforation or transcoelomic spread (through the serosal layer) to the peritoneum. The usual venous drainage of the appendix is via the appendiceal vein on to the ileocolic vein then via the portal vein to the liver.

However, the authors hypothesize that, throughout the natural progression of appendiceal cancer, the appendiceal vein is either obstructed or necrosed; the venous drainage of the appendix is therefore redirected via the parietal peritoneum, which drains into the inferior vena cava, avoiding venous drainage via the liver (*Fig. 1a*). This means that CEA produced by cancers of the appendix will drain into the systemic circulation. Hence, there should be a higher serum CEA level in peripheral venous samples and lower proportion of liver metastases, as the CEA is not filtered through the liver. CEA that has bypassed the liver is then most likely destroyed by macrophages once it reaches the peripheral tissues. This will account for the higher serum CEA level recorded as it has bypassed the liver.

**Fig. 1:**
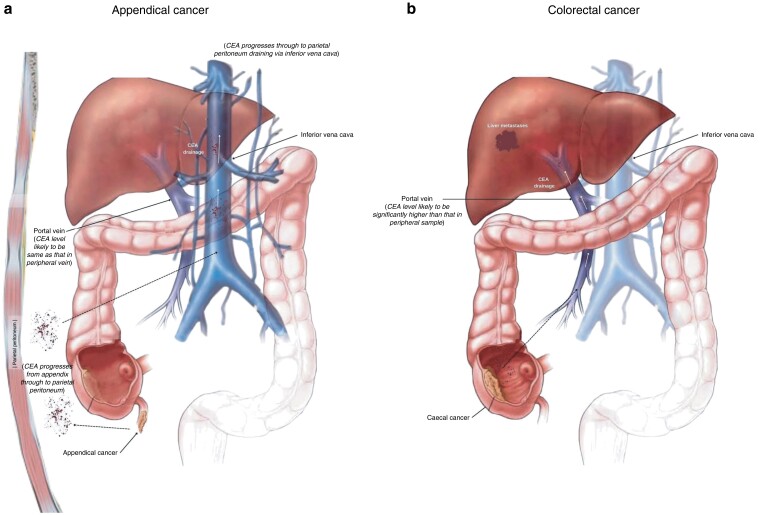
Mechanism of natural progression of appendiceal and colorectal cancer and role of carcinoembryonic antigen in formation of liver metastasis

**a** Appendiceal cancer, and **b** Colorectal cancer as detailed by Lee and Lee^3^.

In terms of colorectal cancers, the colon and rectum drain to the superior and inferior mesenteric vein into the splenic vein then on to the portal vein towards the liver. Colorectal cancer therefore spreads via the portal vein to the liver, along with CEA, causing a higher proportion of liver metastases and proportionally lower, but higher-than-normal serum CEA concentration (*Fig. 1b*). There is a lower serum CEA level in peripheral venous samples from patients with colorectal liver metastases as the liver filters CEA from the colon and rectum.

In the currently available literature, there have been limited studies evaluating the differences between serum levels of CEA in portal and peripheral venous samples from patients with colorectal cancer^4^. Tabuchi *et al*.^5^ has been the only paper to report a significant difference between portal and peripheral venous samples (median_portal_ 26.6 ng/ml, 59.1 per cent over 5 ng/ml *versus* median_peripheral_ 8.1 ng/ml, 33.3 per cent over 5 ng/ml; *P* < 0.050). Unfortunately, these studies have not been replicated in patients with appendiceal cancer.

Based on preliminary sample size calculations, the authors believe that between 24 and 60 patients will be required to show a significant difference (should there be one) between CEA levels in portal and peripheral venous samples. Ethical approval is currently awaited for a study involving prospective enrolment of patients with appendiceal cancer, for peripheral and portal venous blood sampling at the time of index peritonectomy in order to compare serum CEA levels. If there were to be no significant difference in serum CEA concentration between these samples, the authors believe the proposed hypothesis would be supported.

## Acknowledgements

This paper is not based on a previous communication to a society or meeting.

*Disclosure.* The authors declare no conflict of interest.
